# Successful growth of fresh retinoblastoma cells in chorioallantoic membrane

**DOI:** 10.1186/s40942-020-00236-x

**Published:** 2020-07-29

**Authors:** Silvia L. Fialho, Bárbara R. Silvestrini, Juliana Vieira, Mayara R. B. Paiva, Luciana M. Silva, Fernando Chahud, Armando Silva-Cunha, Zelia M. Correa, Rodrigo Jorge

**Affiliations:** 1 Pharmaceutical Research and Development, Ezequiel Dias Foundation, Belo Horizonte, Brazil; 2 Department of Ophthalmology, Ribeirão Preto Medical School, University of São Paulo, Ribeirão Preto, Brazil; 3 Department of Cell Biology, Ezequiel Dias Foundation, Belo Horizonte, Minas Gerais Brazil; 4 Department of Pathology, Ribeirão Preto Medical School, University of São Paulo, Ribeirão Preto, Brazil; 5 School of Pharmacy, Federal University of Minas Gerais, Belo Horizonte, Brazil; 6 Department of Ophthalmology, Wilmer Eye Institute, Johns Hopkins Medicine, Baltimore, MD USA; 7 Department of Ophthalmology, University of Cincinnati College of Medicine, Cincinnati, OH USA

**Keywords:** Retinoblastoma, Tumor models, Chorioallantoic membrane, CAM assay

## Abstract

The authors developed a retinoblastoma model using fresh harvested cells from an enucleated eye that were transplanted in chick embryos (chorioallantoic membrane model). The transplanted embryos were treated with escalating doses of Melphalan. This exploratory model was developed with the goal of testing drug sensitivity. Our findings suggest this tumor model could be employed to personalize treatment for patients with retinoblastoma, especially those with bilateral and more refractory disease.

## Introduction

Retinoblastoma (RB) is a primary neuroectodermal tumor that derives from immature retinoblasts, usually due to a mutation in chromosome 13. It is the most common intraocular cancer that affects children between the ages of 1 and 14 years old [1]. The disease can present as unifocal or multifocal tumors involving one of both eyes. This tumor is considered to be aggressive and grows rapidly potentially destroying the retina in a matter of weeks. Although historically the main therapeutic objective was eradicating the tumor, Reese and Elsworth started to change that by using radiation therapy for globe salvage. The recent development of new local destructive treatments that include cryotherapy, laser photocoagulation, brachytherapy, and intravitreal chemotherapy; and systemic treatments such as intravenous and intra-arterial chemotherapy has increased the chances of eye and sight saving for these children [1].

As these treatment options are more widely used, learning more about individual tumor biology and drug sensitivity is perhaps the next frontier in the management of RB [2–5]. The chicken chorioallantoic membrane model (CAM) has been widely used as an *in*-*ovo/*in vivo model for studies of angiogenesis, metastasis, tumor cell invasion, efficacy and efficiency of antitumor drugs. CAM models for tumor studies specially for RB have utilized established and characterized cell lines such as WERI-Rb1 [5], Y-79, RB 383, and RB 355 [7]. CAM is mainly used because it is highly vascularized and naturally immunodeficient, effective transplantation rate, and quick tumor growth [8–10]. The established cell lines are used because their behavior is known but after multiple passes these immortalized cells are not considered phenotypically representative of RB [3]. For these reasons and scientific data available [10], we used the CAM as a model for in vivo/in-ovo culture of RB cells from fresh enucleated eyes to evaluate its potential to customize treatment for each case.

The purpose of this study is to describe our initial experience using CAM to culture fresh RB tissue harvested from RB enucleated eyes.

## Methods

**Fig. 1:**
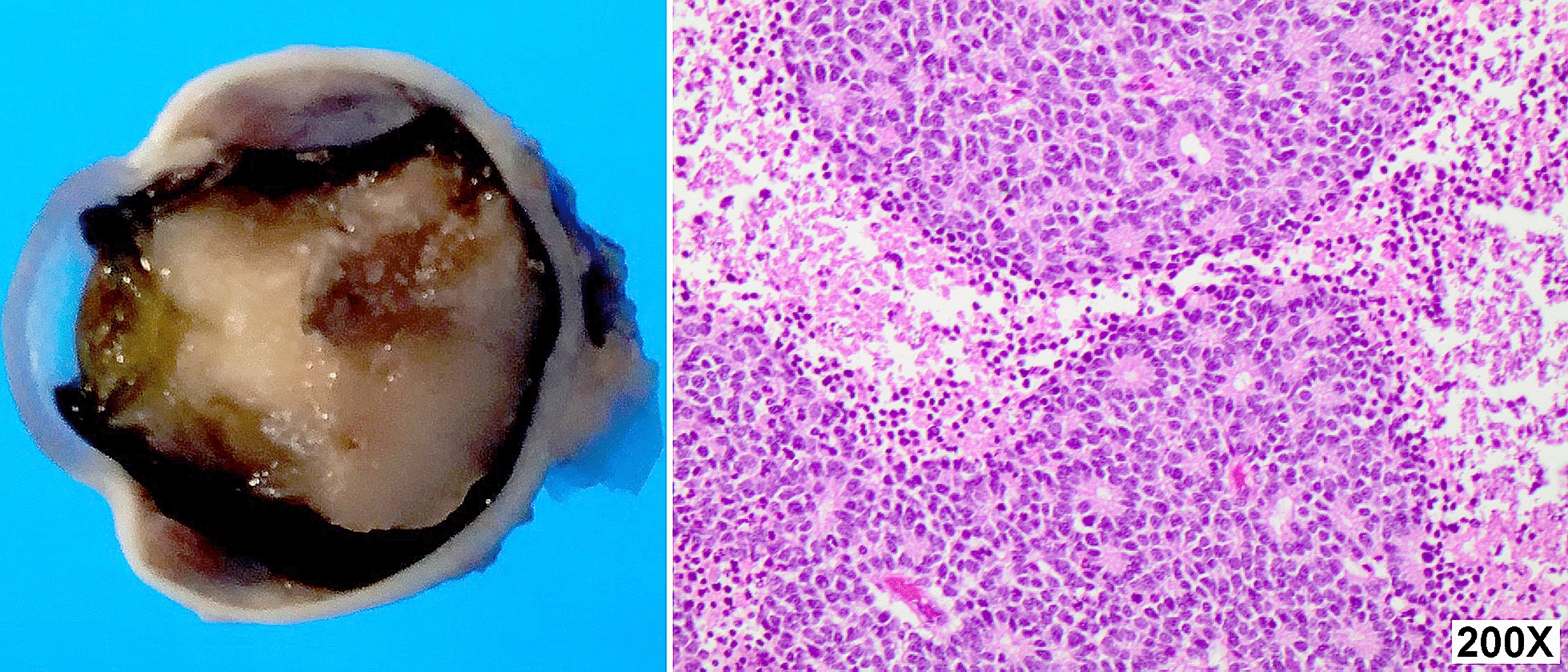
Pathology findings include a large white tumor consistent with retinoblastoma with calcified areas observed on macroscopic evaluation (left). The histology showed a tumor composed predominantly of Flexner-Wintersteiner rosettes surrounded by necrotic cells (right)

The parents of a 6-month-old infant with Group E (International Classification of Intraocular Retinoblastoma) gave written consented to the harvest of fresh tumor sample after enucleation for research and teaching purposes. After performing an enucleation, the fresh globe was opened at the pathology department and tumors cells were retrieved using a scalpel while minimally disrupting the ocular architecture (Fig. 1).

The fragment of fresh tumor was used following a primary culture protocol published by Herrmann et al. in an attempt to expand the RB cells. The expanded tumor culture was used on the CAM assay [9]. This assay was carried out between the 9th and 13th day of embryogenesis, as described by Ribatti et al. [7] For this experiment we used 30 fertilized chicken eggs (n = 5 for each group) randomly assigned to one of 6 groups: 2 control groups—sterile saline administered only (negative control) and RB untreated group—tumor cells transplanted only (positive control), and 4 treatment groups that initially were transplanted with tumor cells and subsequently received Melphalan on the 11th day of this experiment (at escalating concentrations of 20, 30, 40, and 50 µg/ml). Melphalan was the drug of choice based on its current clinical use in RB. On the 13th day (48 h after Melphalan), all the membranes were photographed and analyzed macroscopically.

## Results

**Fig. 2:**
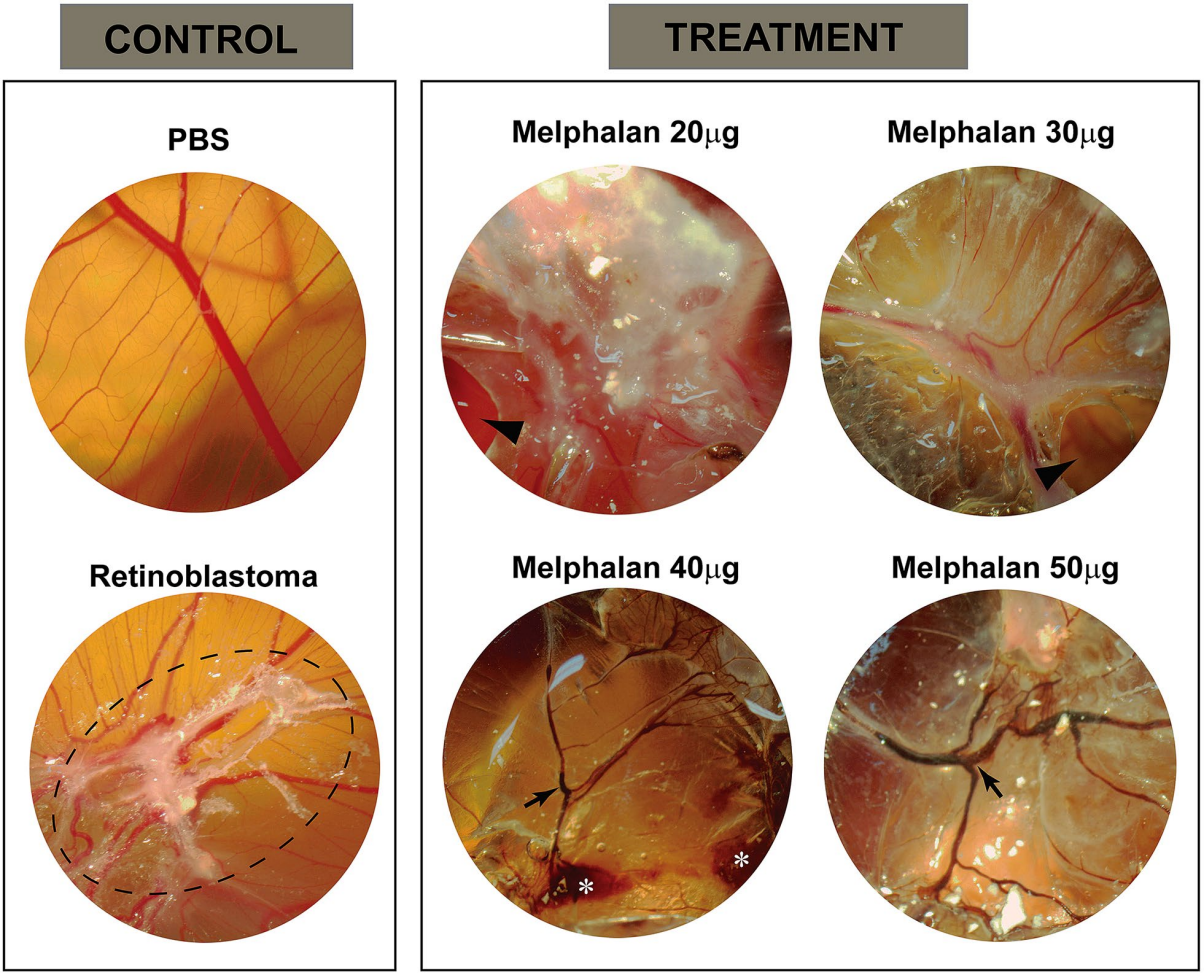
Representative photographs of CAM assay as described. Negative control (PBS) showing the normal vasculature and clear aspect of CAM, positive control (Retinoblastoma) with the development of a white tumor consistent with retinoblastoma (outlined by dotted circle). For the treated samples with different concentrations of Melphalan (20 to 50 µg): Melphalan 20 and 30 µg images show a pronounced tear in the CAM (arrowhead) caused by the accelerated growth of retinoblastoma cells; Melphalan 40 and 50 µg images show hemorrhages (white asterisks), thrombosis and fibrosis (arrows) but with reduced tumor growth

Human RB cells were successfully implanted in CAM for both the control groups and the treatment groups with increasing concentrations of Melphalan. Tumor growth was characterized morphologically by a white mass over the CAM as shown in Fig. 2. Eggs treated with Melphalan showed reduction in the white tumor tissue in a dose-dependent manner (Fig. 2). Tears in the CAM indicated by triangular arrows that are seen in concentrations of 20 and 30 µg/mL could be attributed to tumor shrinkage. Interestingly, at concentrations of 40 and 50 µg/mL, the decrease in tumor volume was accompanied by pronounced vascular insufficiency with hemorrhages (white asterisk), thrombosis (black arrows), and fibrosis. Due to the pilot nature of this study, histopathologic analysis was not performed.

## Discussion

The use of tumor models to study drug resistance in RB has been previously explored [6, 11]. However, most of these studies have looked the fact that established cell lines have shown artifactual changes that don’t correlate closely to the in vivo human tumor biology thus not a good clinical representation of RB behavior in patients [3, 7]. Meanwhile, experimental models of RB using CAM have used American Type Culture Collection (ATCC) cells and not fresh tumor samples harvested after enucleation [6, 11]. Multiple tumor models have been successfully developed from fresh tumor tissue using CAM including ovarian cancer, glioblastoma, nasopharyngeal carcinoma, and renal cell carcinoma [10]. This information prompted us to explore the use of fresh RB cells in CAM to create a model to assess drug response since this is one of the current challenges of treating this tumor. Thus, the preliminary results of our exploratory study demonstrate that it is possible to expand RB cells originated from primary tumor and culture them in CAM in order to test different drugs and identify tumor sensitivity and potential response.

The findings observed using different concentrations of Melphalan point to potential toxicity causing thrombo-hemorrhagic changes when used in higher doses. Despite the conjectural nature of this finding, it correlates to clinical outcomes reported of vascular occlusion and vitreous hemorrhage following intravitreal Melphalan in eyes with RB [12].

Although this model needs to be further studied, it could become a feasible option to personalize patient treatment, especially in bilateral and more refractory cases.
